# T-Synthase Deficiency Enhances Oncogenic Features in Human Colorectal Cancer Cells via Activation of Epithelial-Mesenchymal Transition

**DOI:** 10.1155/2018/9532389

**Published:** 2018-06-21

**Authors:** Xichen Dong, Yuliang Jiang, Jian Liu, Zhe Liu, Tianbo Gao, Guangyu An, Tao Wen

**Affiliations:** ^1^Medical Research Center, Beijing Chao-Yang Hospital, Capital Medical University, Beijing 100020, China; ^2^Department of Oncology, Beijing Chao-Yang Hospital, Capital Medical University, Beijing 100020, China

## Abstract

**Background:**

Immature truncated O-glycans such as Tn antigen are frequently detected in human colorectal cancer (CRC); however, the precise pathological consequences of Tn antigen expression on CRC are unknown. T-synthase is the key enzyme required for biosynthesis of mature O-glycans. Here we investigated the functional roles of Tn antigen expression mediated by T-synthase deficiency in CRC cells.

**Methods:**

To knock out T-synthase, we used CRISPR-Cas9 technology to target C1GALT1, the gene encoding T-synthase, in a CRC cell line (HCT116). Deletion of T-synthase was confirmed by western blotting, and expression of Tn antigen was determined by flow cytometry in HCT116 cells. We then assessed the biological effects of T-synthase deficiency on oncogenic behaviors in HCT116 cells. Furthermore, we analyzed the mechanistic role of T-synthase deficiency in cancer cells by determining the epithelial-mesenchymal transition (EMT) pathway.

**Results:**

We showed that forced knockout of T-synthase in HCT116 cells significantly induced Tn antigen expression, which represented the occurrence of aberrant O-glycosylation. Loss of T-synthase significantly enhanced cell proliferation and adhesion, as well as migration and invasiveness in culture. More importantly, we demonstrated that T-synthase deficiency directly induced classical EMT characteristics in cancer cells. E-cadherin, a typical epithelial cell marker, was markedly decreased in T-synthase knockout HCT 116 cells, accompanied by an enhanced expression of mesenchymal markers including snail and fibronectin (FN).

**Conclusions:**

These findings indicate that T-synthase deficiency in CRC cells not only is responsible for aberrant O-glycosylation, but also triggers the molecular process of EMT pathway, which may translate to increased invasiveness and metastasis in cancers.

## 1. Introduction

**Figure 1:**
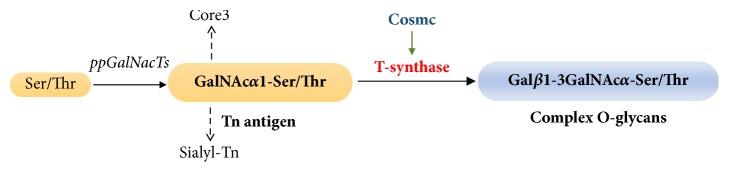
Scheme for the biosynthesis of complex O-glycans. T-synthase is the unique key enzyme during this process.

Mucin type O-glycosylation is the most common posttranslational modification of many transmembrane and secreted glycoproteins. During the process of O-glycosylation, T-synthase (*β*1,3-galactosyltransferase) is the unique essential enzyme that transfers Gal from UDP-Gal to GalNAc*α*1-Ser/Thr (Tn antigen) to form complex O-glycans [1] (Figure 1). Additionally, the expression and activity of T-synthase require an endoplasmic reticulum- (ER-) resident molecular chaperone called Cosmc [2]. Knockout of either T-synthase or Cosmc induces the expression of Tn antigen, indicative of aberrant O-glycosylation. Alterations in O-glycosylation have recently received much attention as a key component in neoplastic progression. Aberrant O-glycosylation appears to correlate with cancer progression, metastasis, and poor prognosis in many epithelial cell-derived cancers such as colorectal cancer (CRC), breast cancer, pancreatic cancer, and cervical cancer [3–7]. Aberrant O-glycosylation may affect cell-cell adhesion and migration, and regulate apoptosis resistance or sensitivity, and eventually participate in tumorigenesis or metastasis [8, 9]. However, the precise mechanistic role of aberrant O-glycosylation has yet to be defined. Interestingly, contrary to most published literature, there recently emerged some controversies regarding the biological consequences of aberrant O-glycosylation caused by T-synthase deficiency in a few malignancies. For instance, Bergstrom et al. reported that although intestinal T-synthase deficient mice developed significant colonic cancers, it was indeed dependent on colitis rather than aberrant O-glycosylation [10]. Song et al. also reported that although loss of T-synthase induced aberrant O-glycosylation, it surprisingly suppressed breast cancer development in mice instead of promoting tumorigenesis [11]. These findings suggest that the functional significance of T-synthase deficiency mediated aberrant O-glycosylation in carcinogenesis needs to be further understood. In this study, we used the CRISPR-Cas9 gene editing technology to knock out T-synthase in a colorectal cancer cell line (HCT116) and resultantly induced aberrant O-glycosylation characterized by the Tn antigen expression in cells. We found that T-synthase deficiency promoted directly the oncogenic properties of cancer cells, which may be attributed to activation of EMT signaling pathway.

## 2. Materials and Methods

### 2.1. Cell Culture

Human colorectal carcinoma cell line HCT116 and human embryonic kidney cell HEK293T were obtained from American Type Culture Collection (ATCC, USA) and maintained according to ATCC guidelines. HCT116 cells were cultured in McCoy's 5A medium (Gibco) and HEK293T cells were cultured in DMEM medium (Sigma). Both mediums were supplemented with 10% fetal bovine serum and 1% penicillin/streptomycin. All cells were cultured at 37°C in a humidified incubator with 5% CO_2_. HCT116 cells were infected with LentiCRISPR v2 virus constructed for targeted genes and maintained in media containing 2 *μ*g/ml puromycin (Gibco).

### 2.2. Plasmid Construction

To knock out T-synthase in HCT116 cells, we designed one pair of single guide RNA (sgRNA) sequences (F: 5′-CACCGATCCTATTGCTGATCCACAG-3′ R: 5′-AAACCTGTGGATCAGCAATAGGATC-3′) specifically targeting C1GALT1 that encodes T-synthase using the design tool from the Feng Zhang's Lab. The sgRNA sequences were annealed and then cloned into the LentiCRISPR v2 vector (a gift from Feng Zhang; Addgene plasmid #52961) predigested with the BsmBI enzyme (New England Biolabs) as previously described [12]. The construct was confirmed by sequencing (Sangon Biotech, China).

### 2.3. Lentivirus Packaging and Infection of Cells

The constructed sgRNA producing lentiviral vector and the psPAX2 and pMD2.G plasmids were cotransfected into HEK293T cells using lipofectamine 3000 (Invitrogen) following the manufacturer's instructions. The generated viruses were harvested at 48 hours and 72 hours after transfection and then infected HCT116 cells in a 6-well plate (Corning) with approximately 50% confluence. At 72 hours after infection, the culture medium was replaced by complete medium containing 2 *μ*g/ml puromycin (Gibco) and the cells were screened for 7 days before western blot identification.

### 2.4. Flow Cytometry Analysis

The expression of Tn antigen after T-synthase knockout in cells was analyzed by a Flow Cytometer (Canto II, BD Bioscience). In brief, 1×10^5^ cells were suspended in cold PBS and incubated with 10 *μ*g/mL mouse anti-Tn IgM mAb (kindly provided by Dr. Tongzhong Ju, Emory University, Atlanta) or mouse IgM isotype-antibody as a control (Santa Cruz), for 2 hours at 4°C. Then the cells were washed with cold PBS three times and incubated with PE-labeled goat anti-mouse IgM secondary antibody (BD, 562033) for 1 hour at 4°C. After washing with PBS three times, the cells were analyzed by flow cytometry (Canto II, BD Bioscience).

### 2.5. Cell Proliferation Assay

A Real-Time Cell Analyzer (RTCA, ACEA Biosciences) was used to assess the cell proliferation ability as previously reported [13]. Briefly, 5000 cells in 200 *μ*l complete medium were seeded into each well of the E-plate (ACEA Biosciences) with 4 replicates. After connecting the E-plate with analyzer, the cell index in a 160-hour period was recorded automatically every 15 min interval by RTCA software.

### 2.6. Cell Migration and Invasion Assay

Transwell assay was performed to examine the cell migration ability. 2×10^5^ cells were suspended in 200 *μ*L medium with no FBS and seeded into a transwell insert (Costar®, 3422, USA).The carrier chamber was added with 500 *μ*L complete medium containing 10% FBS. After 24 hours, the migrating cells were fixed with 100% ethanol for 30 min and stained with 0.1% crystal violet for 30 min. After washing with PBS for twice, the nonmigrating cells on the insert were wiped off prior to microscopic observation. The migrating cells under 4 random views were counted. The invasion assay was performed the same as migration except that the transwell insert was precoated with matrigel (BD).

### 2.7. Adhesion Assay

Cell adhesion ability was examined by CCK-8 method (Dojindo). Briefly, 4×10^4^ cells suspended in 100 *μ*L complete medium were seeded into each well of a matrigel-coated 96-well plate (Corning). After adhesion for 5 min, the floating cells were gently washed off with PBS.10 *μ*L CCK-8 reagent was then added to each well of the plate and incubated for 2 hours at 37°C with 5%CO_2_. The assay was performed in triplicate. The absorbance was measured at a wavelength of 450 nm by a Varioskan Flash (Thermo Scientific).

### 2.8. Western Blot Analysis

Total protein lysates were prepared with RIPA lysis buffer (EnoGene, E1WP108) supplemented with 1 mM protease inhibitor cocktail (EnoGene) and 1 mM phenylmethylsulfonyl fluoride (PMSF, EnoGene). The supernatants were collected after centrifuging at 12,000×g for 10 min at 4°C. The protein concentration was measured by BCA Protein Assay Kit (Thermo Fischer). Samples in loading buffer were boiled for 10 min at 95°C. Proteins were separated by SDS PAGE and transferred onto a PVDF membrane (Millipore). The membrane was blocked with 5% skim milk (BD, 232100) and probed with following primary antibodies against snail, E-cadherin, N-cadherin, Fibronectin (FN), and GAPDH from Cell Signaling Technologies and T-synthase from Santa Cruz. After washing with TBST 3 times, the membrane was subsequently incubated with HRP-conjugated secondary antibody (1:8000, ZSGB-BIO). After the final washing with TBST 3 times, the signal was detected using chemiluminescent HRP substrate (Millipore) on the Bio-Rad imaging system (Bio-Rad ChemiDoc MP, 1708195).

### 2.9. Statistical Analysis

Data analysis was performed by SPSS 23 software (SPSS Inc., Chicago, IL, USA). Statistical differences were analyzed by Student's* t*-test (unpaired, 2-tailed) and p<0.05 was considered significant. Independent experiments were repeated at least three times and the data were represented as mean± SD.

## 3. Results

### 3.1. Induction of Tn Expression by Deleting T-Synthase in HCT116 Cells

**Figure 2:**
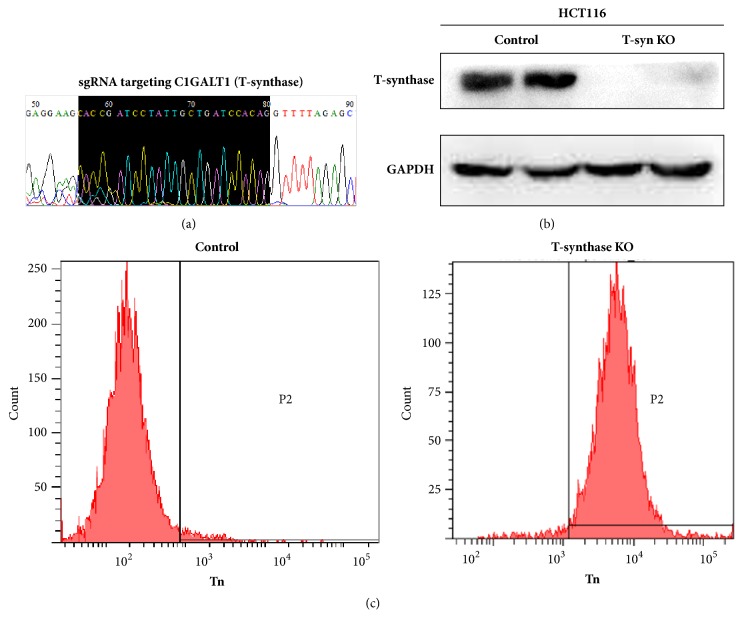
Forced deletion of T-synthase in HCT116 cells via the CRISP/CAS9 method. (a) One pair of single guide RNA (sgRNA) was designed to specifically target C1GALT1 that encodes T-synthase. (2) Western blot analysis was performed to confirm deletion of T-synthase in cells. (3) Tn antigen expression, indicative of aberrant O-glycosylation, was determined by flow cytometry in cells.

We used CRISPR-Cas9 to disrupt enzymatic reactions required for the extension of O-glycans by deleting T-synthase in HCT116 cells. Knockout of T-synthase was confirmed by western blot (Figure 2). Then we measured Tn antigen expression by FACS. The results showed that HCT116 cells showed abundantly Tn expression after T-synthase knockout, indicative of induction of aberrant O-glycosylation. There was no Tn staining in the control cells that were infected with control virus and still had T-synthase expression (Figure 2(c)).

### 3.2. T-Synthase Deficiency Mediated Aberrant O-Glycosylation Enhances Malignant Behaviors

**Figure 3:**
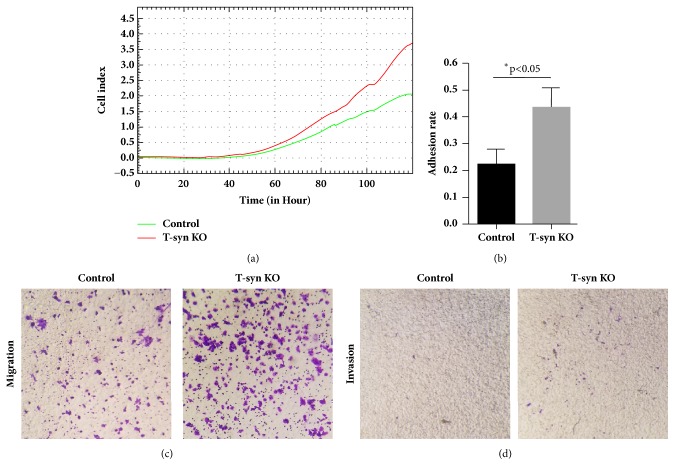
T-synthase deficiency promotes oncogenic features in HCT116 cells. (a) T-synthase KO cells showed an enhanced proliferation rate in contrast to the control. (b) Cell adhesion, (c) migration, and (d) invasion in T-synthase KO cells were more pronounced than in control cells. Representative results from triplicate experiments were shown.

We then evaluated whether aberrant O-glycosylation induced by T-synthase deficiency imposed a direct influence on malignant properties in cancer cells. Cell proliferation, adhesion, migration, and invasiveness were assessed, respectively. We used RTCA to monitor in real time the cell proliferation rate and observed that T-synthase deficient cells (Tn-positive) had an enhanced proliferation rate compared to the control cells (Tn-negative) (Figure 3(a)). Adhesion to matrix is the first step in cancer cell metastasis [14]. Therefore, prior to migration and invasion assays, we first assessed the influence of aberrant O-glycosylation on cell adhesion capacity. It showed that the cell adhesion activity in T-synthase deficiency cells (Tn-positive) was significantly higher in comparison with that in control cells (Figure 3(b)). To investigate whether aberrant O-glycosylation might regulate cell motility, we performed transwell migration and matrigel invasion assays. Our data showed that T-synthase knockout significantly increased migration and invasion of HCT116 cells (Figures 3(c) and 3(d)). Together, these results indicate that aberrant O-glycosylation caused by T-synthase deficiency can enhance malignant phenotypes of colon cancer cells in vitro.

### 3.3. T-Synthase Deficiency Activates the EMT Pathway

**Figure 4:**
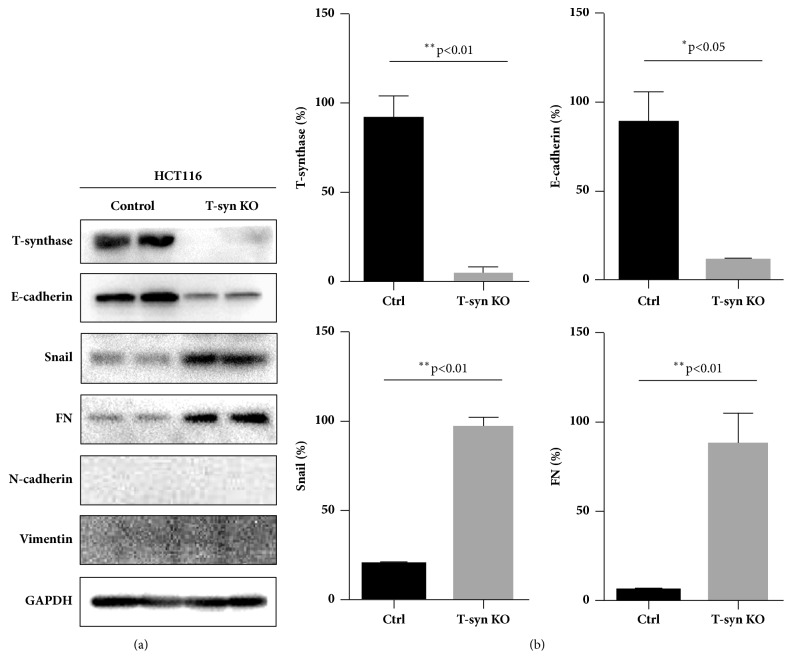
Activation of the EMT process in T-synthase KO cells. (a) Western blot analysis showed that E-cadherin was decreased significantly, along with an upregulated expression of snail and FN in T-synthase KO cells. N-cadherin and vimentin were not detectable. Representative results from triplicate experiments were shown. (b) Signal intensities were normalized, and values were quantitatively shown as relative intensity (mean ± SD) for T-synthase, E-cadherin, snail, and FN.

We further checked how T-synthase deficiency exerts biological effects on the cellular behaviors. The EMT process is one of the key signaling pathways in cancer progression and metastasis [15]. Therefore, we checked whether T-synthase deficiency had an influence on EMT pathway. Western blot analysis showed that the epithelial marker E-cadherin was significantly decreased in Tn-positive 116 cells. Although N-cadherin and Vimentin were not detected in Tn-positive or Tn-negative 116 cells, other mesenchymal markers, such as snail and FN, were remarkably upregulated in Tn-positive cells (Figure 4), thereby indicating that the cancer cells underwent an EMT event after T-synthase knockout. These results suggest that aberrant O-glycosylation caused by T-synthase deficiency may activate EMT signaling pathway.

## 4. Discussion

Expression of the truncated O-glycans such as Tn antigen represents aberrant O-glycosylation, which is closely associated with many epithelial-derived cancers including CRC, breast cancer, and pancreatic cancer. Although Tn antigen is frequently detected in CRC tissues, the biological significance of its expression is still not fully understood. Studies have reported that Tn antigen expression correlates with cancer progression and metastasis in many malignancies [16]; however, it is not yet known whether Tn antigen acts as a causal factor for cellular transformation during tumorigenesis. Moreover, there are indeed some contradictory findings regarding the functional role of Tn antigen in cancer progression and metastasis. For example, Radhakrishnan et al. demonstrated that Tn antigen expression in pancreatic cancer cell lines markedly induced classical oncogenic features [17]; it has also been reported that expression of Tn antigen in breast cancer cells produced diverse biologic and pathologic consequences influencing growth and survival of the cells and their invasion and metastasis ability [18]. Conversely, Bergstrom et al. surprisingly showed that Tn antigen was independent of cancer progression in a murine model of colorectal cancer; intestinal inflammation rather than aberrant O-glycosylation led to the eventual tumorigenesis [19]. Therefore, the precise functional role of Tn antigen (aberrant O-glycosylation) remains to be fully elucidated.

Normally, the Tn antigen is modified to form elongated and complex O-glycans by a specific galactosyltransferase (T-synthase) in the Golgi apparatus of cells [20]. Interestingly, the activity and expression of T-synthase are found to be dependent on a unique chaperone called Cosmc [21]. Either T-synthase or Cosmc dysfunction can induce Tn antigen expression [22]. In addition, there are also other possible factors that may lead to the Tn antigen expression, such as relocation of GalNAc-transferases from Golgi to endoplasmic reticulum. Among these mechanisms, T-synthase dysfunction is the most prevailing [23]. Therefore, we used a precise gene editing method (CRISPR-Cas9) to knock out T-synthase in CRC cells, which can disrupt the gene expression by editing the gene at the DNA level and perform gene deletion with high efficiency [24]. We validated that T-synthase deficiency induced significant occurrence of aberrant O-glycosylation, characterized by Tn antigen expression in cancer cells. We thereby obtained the matched Tn-positive and Tn-negative CRC cells originated from the same parent cells, which enabled us to analyze the functional role of Tn antigen in cancer cells. We first confirmed that Tn antigen expression caused by T-synthase deficiency significantly enhanced oncogenic features such as cell proliferation, cell adhesion, migration, and invasion in cancer cells. These findings provide direct evidence that aberrant O-glycosylation, characterized by Tn antigen expression, contributes to cancer progression and metastasis in CRC. Tn antigen appears to serve not only as a tumor carbohydrate marker but also as a potential target for therapeutic intervention [25, 26]. To the best of our knowledge, it is the first report to reveal the biological consequences of Tn antigen expression caused by T-synthase deficiency on CRC cells.

We next sought to elucidate how Tn antigen expression affects oncogenic behaviors in cancer cells. It is known that expression of Tn antigen represents alterations in O-glycosylation of many glycoproteins, and these alterations may further affect cellular signaling, metabolism, or receptor function. Expression of the Tn antigen may lead to or reflect the altered pathophysiological processes [27]. However, because Tn antigen produces global effects on the O-glycosylation of many proteins, it is not straightforward to decipher the individual O-glycoprotein during this process. Therefore, we intended to decipher which key pathways were most affected by Tn antigen expression caused by T-synthase deficiency. In view of the many documented examples demonstrating the associations between Tn antigen and cancer progression and metastasis, we assumed that Tn antigen mediated aberrant O-glycosylation was involved in epithelial-mesenchymal transition (EMT) process, because EMT process plays a key role in cancer progression and metastasis [28, 29]. In this study, we found that T-synthase deficiency significantly activated the EMT pathway, demonstrated by a reduced expression of epithelial cell marker E-cadherin and an enhanced expression of mesenchymal cell markers such as snail and fibronectin (FN). A classical mesenchymal marker N-cadherin was not detected in HCT116 cells; however, the changes of other mesenchymal markers, snail and FN, were sufficient to assess the EMT process.

In summary, we show that T-synthase deficiency can induce aberrant O-glycosylation in cells, which in turn promotes oncogenic features in cancer cells via the activation of EMT process. Our study shows that aberrant O-glycosylation plays a contributory role in cancer progression and metastasis. Further investigations are required to explore other possible mechanisms or pathways.

## Data Availability

The data used to support the findings of this study are available from the corresponding author upon request.
